# Workflow interruption and nurses’ mental workload in electronic health record tasks: An observational study

**DOI:** 10.1186/s12912-023-01209-9

**Published:** 2023-03-09

**Authors:** Yawei Shan, Jing Shang, Yan Yan, Xuchun Ye

**Affiliations:** 1 School of Nursing, Naval Medical University, No. 800 Xiangyin Road, Shanghai, 200433 China; 2 School of Nursing, Shanghai University of Traditional Chinese Medicine, Shanghai, China

**Keywords:** Nursing interruption, Mental workload, Task performance, Electronic nursing record, Observational study

## Abstract

**Background:**

Workflow interruptions are common in modern work systems. Electronic health record (EHR) tasks are typical tasks involving human–machine interactions in nursing care, but few studies have examined interruptions and nurses’ mental workload in the tasks. Therefore, this study aims to investigate how frequent interruptions and multilevel factors affect nurses’ mental workload and performance in EHR tasks.

**Methods:**

A prospective observational study was conducted in a tertiary hospital providing specialist and sub-specialist care from June 1^st^ to October 31^st^, 2021. An observer documented nurses’ EHR task interruptions, reactions and performance (errors and near errors) during one-shift observation sessions. Questionnaires were administered at the end of the electronic health record task observation to measure nurses’ mental workload for the electronic health record tasks, task difficulty, system usability, professional experience, professional competency, and self-efficacy. Path analysis was used to test a hypothetical model.

**Results:**

In 145 shift observations, 2871 interruptions occurred, and the mean task duration was 84.69 (SD 56.68) minutes per shift. The incidence of error or near error was 158, while 68.35% of errors were self-corrected. The total mean mental workload level was 44.57 (SD 14.08). A path analysis model with adequate fit indices is presented. There was a relationship among concurrent multitasking, task switching and task time. Task time, task difficulty and system usability had direct effects on mental workload. Task performance was influenced by mental workload and professional title. Negative affect mediated the path from task performance to mental workload.

**Conclusions:**

Nursing interruptions occur frequently in EHR tasks, come from different sources and may lead to elevated mental workload and negative outcomes. By exploring the variables related to mental workload and performance, we offer a new perspective on quality improvement strategies. Reducing harmful interruptions to decrease task time can avoid negative outcomes. Training nurses to cope with interruptions and improve competency in EHR implementation and task operation has the potential to decrease nurses’ mental workload and improve task performance. Moreover, improving system usability is beneficial to nurses to mitigate mental workload.

**Supplementary Information:**

The online version contains supplementary material available at 10.1186/s12912-023-01209-9.

## Background

Workflow interruptions are common in modern work systems that include complicated elements such as task demands, interpersonal exchange, information technologies and even conflicting information [1]. Workflow interruptions in healthcare have received considerable attention, and some studies have suggested that they are associated with compromised working efficiency, error and patient safety [2]. In this study, we defined nursing interruptions as external behaviours that might distract nurses’ attention from the initial task, requiring task switching or concurrent multitasking, which might eventually affect the work continuity and mental workload of nurses [3]. While most studies have focused on the negative aspects of interruptions, others have presented a broader view, acknowledging that some interruptions, especially those leading to nursing task switching, might be beneficial and necessary for the quality of the task [4, 5]. Hence, the consequences of interruptions warrant further research.

A literature search showed that existing nursing interruption studies have mainly focused on the whole workflow of nursing care, lacking specific attention to certain tasks [6, 7]. Different nursing tasks have unique characteristics that differentiate them from each other either in terms of the types of interruptions, nurses’ reactions to these interruptions, or the influence on nurses’ mental workload and task error. Apart from very few studies focused on the interruption of medication administration, which is considered a priority in nursing care [2], there is a lack of investigations on specific nursing tasks. With the ever-growing adoption of health information technology, electronic health record systems (EHRs), as a digital form of patient health information designed to improve health service delivery and care quality [8], play an important role in the information management of nursing care. However, interruptions in EHR tasks are quite common, as EHR tasks are considered one of the lowest priorities in nursing care under the modern nursing emphasis on being "human oriented"; hence, there is a dearth of studies examining EHR tasks [2].

EHR tasks are typical tasks involving human–machine interactions in nursing care, and EHRs play an integral role in modern care to facilitate documentation practices such as assessment, care planning and evaluation [9]. Furthermore, EHRs are often used to provide evidence of the quality of care that has been delivered, thus protecting nurses in case of complaints. Although evidence on the effect of interruptions on nursing care has been produced for two decades [1], little attention has been given to the sequelae of interruptions regarding the functioning of nurses’ EHR tasks and their mental workload in natural clinical settings. Studies have called for action to cope with workflow interruptions to reduce task error rather than blaming clinicians and urging them to try harder [10, 11]; thus, to improve clinical quality and nurses’ psychological health, it is worthwhile to investigate the source of interruptions and the consequences for nurses’ behaviour alteration during EHR tasks, as well as to understand the influence of interruptions on cognitive processes and task performance.

Emerging insights from relevant evidence could provide theoretical and methodological references for this study. According to previous studies, the influence of interruptions on nurses’ task cognition is controversial. A study by Matthias Weigl et al. [6] showed that workflow interruptions had a negative impact on physicians and nurses in emergency departments; that is, high rates of interruptions were associated with a lower level of situational awareness. In a study by McCurdie et al. [5, 12], some kinds of interruptions, especially those providing necessary informational content for the initial task, contributed to improving nurses’ cognitive processes, allowing them to achieve fast and efficient patient care [4]. Therefore, the source of interruptions and responses should be analysed to determine the effects of interruptions in EHR tasks.

The hypothetical framework in this study involved several interrelated factors according to the cognitive load framework in researches concluded by Jahns [13], including the inputs (mainly the source of interruption), the behaviour alteration (including task switching and concurrent multitasking), and the outputs (including mental workload and task error). Based on prior research [4, 14], we classified nurses’ reactions to workflow interruptions as task switching or concurrent multitasking. Task switching is defined as the response when ongoing tasks are interrupted by an unexpected event with the consequence of discontinuation of the primary task or task switching from this primary task; on the other hand, concurrent multitasking is defined as the simultaneous performance of the new task and the primary task, and it could turn into task switching when there are limitations on cognitive resources or on the time the task should be completed. These two responses rely on different neuronal pathways and induce different consequences [15]. Drawing upon the previous studies, working time accelerates with high frequency of interruptions, thus increases the level of perceived workload and compromise task performance [16]. As such, we proposed the first hypothesis.

- Hypothesis 1: there is a connection among concurrent multitasking, task switching and task time in interruptive context, thus increasing mental workload and afterwards negatively influencing task performance.

To analyse the influence of other factors including task demand, technology and individual characteristics on nurses’ behavioural alterations in EHR tasks and their general cognitive processes, a comprehensive theoretical framework to guide this study should be referenced. According to the human factors and ergonomics perspective of mental workload [13], mental workload is a multidimensional concept reflecting work behaviour, effort and performance in complex and dynamic situations [17]; it is defined as the amount of thinking, level of cognitive demand, or amount of thought processing effort required by the worker to meet the environmental, physical and temporal demands of a certain task [18]. In this framework, environmental factors (interruptions), along with the factors of task demand (task time, task difficulty) [19], technology (system usability) [20] and individual characteristics (professional competency, self-efficacy and mood), are the key variables that influence psychophysiological responses, workload modification and performance in human–machine interaction tasks [21].

According to the technology acceptance model (TAM) [22] and study on the influence of individual characteristics on the evaluation of hospital information system from Chen [23], high level of professional experience improves self-efficacy on the technology and task, and is negatively related to mental workload and could increase task performance. Hence, we proposed the second hypothesis. In terms of the negative psychological reaction, the influence of personal affect on the level of perceived workload is well established [24], thus influencing consequently, a decline in task performance in physically and emotionally challenging context [25]. Moreover, according to the theory of human based dynamics of mental workload in complicated systems [21], the decreased task performance increases the performance pressure leading to negative affect. As such, we proposed the third hypothesis.

- Hypothesis 2: self-efficacy plays a mediating role in the relationship between professional experience and mental workload.

- Hypothesis 3: negative affect can increase nurses’ mental workload and task performance. In turn, task performance will influence personal affect.

In the context of human factors and ergonomics perspective [13] and technology acceptance model [22], task difficulty and system usability act as task demand and job characteristics that influence nurses’ mental workload. As reported in a study of evaluation of different system interface designs, EHRs with enhanced usability appears to be associated with better physician cognitive workload and performance [26]. Moreover, a task with high level of difficulty could result in insufficient personal resources to meet task-related requirements under the limited strength model [27], nurses should motivate more cognitive resource in difficult tasks. In addition, studies have broadly elucidated that the professional experience has positive influence on nurses’ perceived task difficulty by enhancing cognitive resources and skills [28]. Concerning the relationship between system usability and task difficulty in EHR tasks, low level of system usability hinders the task operation, and increases nurses’ perceived task difficulty [29]. Drawing upon the theories and previous studies, we asserted the fourth and fifth hypotheses as follows.

- Hypothesis 4: high level of task difficulty and lower level of system usability can increase nurses’ mental workload directly.

- Hypothesis 5: task difficulty plays a mediating role in the relationship between system usability and mental workload, as well as the relationship between self-efficacy and mental workload.

Therefore, a hypothetical framework to describe and understand nurses’ general cognitive processes in EHR tasks in dynamic and interruptive clinical settings was proposed (Appendix Fig. 1). The study was conducted with the following aims: (1) to observe and describe workflow interruptions, nurses’ responses and performance during EHR tasks and (2) to study how interruptions, task time, task difficulty, system usability, and individual characteristics influence nurses’ mental workload and task performance based on the hypothetical model.

## Methods

### Study design, setting and participants

A prospective observational method approach was employed in this study, and naturalistic observation and questionnaire investigation were conducted. The study setting was a tertiary teaching hospital which provided specialist and sub-specialist care and was affiliated to a medical university). The hospital was divided into four clinical areas: the internal medicine department, surgical medicine department, emergency room and intensive care unit. Nurses involved in EHR tasks in the internal medicine and surgical medicine departments were observed and investigated in our study because the work patterns of the emergency room and intensive care unit were different. The observations were conducted at nurse stations where the desktop computers were located, and usually nurses performed EHR tasks there even though tablets were introduced. The EHR system in this hospital had been installed only one year ago as the previous one was unsuitable for paperless office. Nurses completed the assessment and care plan using a template in the EHR, and could only view documents completed by other healthcare professionals. According to Cohen’s sample size recommendation [30] and using G*Power [31] and the 10 times rule of thumb, a minimum of 98 participants were required for this study.

### Data collection procedures

Non-participatory observation and investigation were conducted from June 1st to October 31st, 2021. A total of 152 nurses (response rate 98.70%) were initially permitted to participate and were observed in all EHR tasks during their shifts (8 h). Day shifts were scheduled from 8:00 A.M. to 3:30 P.M. or 8:00 A.M. to 5:00 P.M., and the night shift was from 5:00 P.M. to 1:00 A.M. Questionnaires including several scales were distributed at the end of the EHR task observation to measure nurses’ mental workload for the task and to explore the potential influencing factors on mental workload and task performance. Initial permission was obtained from various department heads and hospital administrators before the study was conducted. Prior to each observation, individual nurses were informed about the study objectives and procedure, and written consent was obtained.

## Measurements

### Sociodemographic characteristics

A sociodemographic questionnaire was designed to collect information on characteristics, including gender, age, marriage status, education, years of clinical practice, professional title, and practice department.

### Observation of EHR tasks and interruptions

A self-designed observational tool was applied to document EHR tasks (e.g., assessment, care planning and evaluation) and concurrently classify the source and content of each observed interruption event (e.g., interruptions from the environment, patients, nurses’ colleagues, nurses themselves, patients’ family members, doctors, technical malfunctions, information impediments, telephone/equipment alarms, clinical teaching) and reaction (task switching or concurrent multitasking) over time. The self-designed observational tool was firstly drafted based on a three-month field observation and interviews of twelves nurses, and a literature review on studies of nursing workflow interruptions to identify the core elements and classification of interruptions. Expert consultation with ten health care professionals for content revision and validity was then conducted. A twelves-nurse pilot observation was carried out to test the revised version of the observational tool and then developed the final version (Appendix Observational tool for EHR tasks 1). The total EHR task time was calculated from the beginning to the end of the process (one shift), while medicine administration or other usual care with fixed times was observed and recorded with free text and then eliminated during calculation by observer.

### Observation and measure of task performance

Performance was measured based on the incidence of errors and near errors. Potential error and near errors included (1) the input of incorrect patient data or diagnosis; (2) the use of incorrect nursing terminology; (3) the use of incorrect templates; (4) the unexpected exit from the EHR due to maloperation; and (5) a slow log-in processes. Drawing on the definition of near error in human factors and ergonomics research [32], a near error in EHR task is an unintentional incident in which no real mistake is made though self-checking and revision with prior completed assessment or care plan, but when there is a slight shift in time or position, error may occur easily. An index of performance was calculated, with an error counted as 2 points and a near error counted as 1 point. The total point in one shift represents the nurse’s index of performance.

### Measurement of mental workload

Mental workload data were obtained using the Chinese version of the National Aeronautics and Space Administration Task Load Index (NASA-TLX) [33]. The NASA-TLX is a well-validated and widely used measure in human factors and ergonomics that comprises six subscales or dimensions regarding different aspects of workload (mental demands, physical demands, temporal demands, performance, effort, and frustration). The Chinese version was translated by Liang L et al. [34]; in this version, the items are rated on a 20-point bipolar scale that ranges from 0 to 100. For five of the six dimensions, i.e., mental demands, physical demands, temporal demands, effort, and frustration, a score of 0 indicates the lowest task load; however, the performance dimension is reverse-scored, with 0 indicating the most successful performance of the task and the highest level of satisfaction with one’s performance. The Cronbach’s ɑ of the total Chinese version of the scale is 0.782 [34]. In this study, we used the total (mean) mental workload score rather than the weighted workload score.

### Measurement of task difficulty

The difficulty of EHR tasks was evaluated by the Chinese version of the Difficulty Index of Clinical Nursing Operation Technique, which was developed by Liyan Liu [35]. The instrument consists of 11 items rated on a 5-point Likert scale, ranging from “lowest” to “highest”. The subscales of this instrument consist of the subordinate concepts of task complexity, operator requirements, operating conditions, task intensity and operational risk.

### Measurement of system usability

The usability of EHR system was measured using a system usability scale originally created by John Brooke in 1986, which is a widely used self-report scale regarding to technique products including hardware, software, mobile devices, websites and applications, ranging from 1 (strongly disagree) to 5 (strongly agree). Five statements are positively formulated (items with odd numbers), and 5 statements are negatively formulated (items with even numbers) [36].

### Measurement ofprofessional competency

Professional competency was measured using the Chinese version of the Competency Inventory for Registered Nurses developed by Ming Liu et al. [37]. The subscales of the instrument consist of the subordinate concepts of critical thinking and research aptitude, clinical care, leadership, interpersonal relationships, legal/ethical practice, professional development, and teaching coaching. The scale has 58 items, and the responses are rated on a 5-point Likert scale, with 4 representing the highest level of the concept. At the time of the scale’s development, the total scale internal consistency of Cronbach’s ɑ was 0.91, and the Cronbach’s ɑ coefficients of the dimensions ranged from 0.77 to 0.87. In this study, the subscales of critical thinking and research aptitude and clinical care, which include 18 items, were employed.

### Measurement ofself-efficacy

Self-efficacy was evaluated with the Chinese version of the General Self-Efficacy Scale [38], which comprises 10 items rated on a 4-point Likert scale, ranging from “completely incorrect” to “completely correct”. The total score of the scale ranges from 10 to 40, with higher scores indicating higher confidence. The Cronbach’s ɑ was 0.883 in the present study [39].

### Measurement ofemotion

Nurses’ emotions during the EHR task were measured with the Chinese version of the Positive and Negative Affect Scale [40], a self-reported instrument comprising positive and negative domains with 10 items each. Responses are rated on a 5-point Likert scale ranging from 1 (very slightly or not at all) to 5 (extremely), with higher scores indicating a higher level of either positive or negative affect. The Cronbach’s ɑ coefficients were 0.93 and 0.90 for the positive and negative domains, respectively [41].

### Validity and reliability

The psychometric properties of the measurement tools have been described above. Moreover, the observer (first author) was a registered nurse who had 6 years of clinical practice experience and was a senior lecturer at a medical university. To ensure familiarity with the EHR tasks, the observer was familiar with each patient’s courses of disease, diagnosis, treatment, nursing care and prognosis. Additionally, to increase sensitivity to task errors, the observer reviewed 234 EHRs randomly selected from the last quarter in this hospital under the guidance of staff of the quality control department.

### Statistical analysis

The counts of observed interruptions were calculated as rates per shift of observation overall. For each interruption, the source and the nurses’ reaction (task switching or concurrent multitasking) over a certain time period were calculated in terms of the frequency. Statistical analysis was carried out with SPSS 21.0 software, and *p* < 0.05 (two-tailed) was considered statistically significant. Descriptive statistics, including the number (n), percentage (%), mean and standard deviation (SD), were used to analyse the demographics, interruptions, task time, task difficulty, system usability, professional experience (title and competency), self-efficacy, mental workload, and task performance (error and near error). Tests for normality and equal variance for all the study variables were performed using Shapiro–Wilk’s and Bartlett tests. Pearson's correlation was applied to evaluate correlations between variables according to the hypothetical model. Path analysis was used with the maximum-likelihood method implemented in SPSS Amos v23.0 software (IBM Corp) to examine the hypotheses [42]. The fit indices were examined to determine the appropriated model, including χ^2^, root mean square error of approximation (RMSEA), goodness-of-fit index (GFI), adjusted goodness-of-fit index (AGFI), comparative fit index (CFI), Tucker-Lewis index (TLI), normed fit index (NFI) and incremental fit index (IFI). The path coefficient estimated the intensity between two study variables and was analysed using a standardized regression coefficient (β weight).

## Results

### Descriptive statistics of demographic characteristics

In our study, the total number of nurses involved in EHR tasks in the internal medicine and surgical medicine departments was 198; 44 nurses were absent during the period of our investigation for anti-epidemic tasks outside of the hospital. Therefore, the targeted sample was 154. With consent of the participants, 152 observation shifts with 152 nurses were conducted, and complete data were collected for 145 of the shifts, of which 32.41% were night shifts. Overall, 138 (95.17%) of the observed nurses were female, 48.28% ranged in age from 20–30 years, and 31.72% had 6–10 years of clinical practice. The demographic characteristics of the participants are shown in Table 1.

**Table 1:** Sociodemographic and occupational characteristics of the nurses (*n* = 145)

Characteristic		Number	Percentage (%)
Overall		145	100.00
Gender	Man	7	4.83
	Woman	138	95.17
Age, y	20–30	70	48.28
	31–40	56	38.62
	> 40	19	13.10
Marriage status	Unmarried	60	41.38
	Married	85	58.62
Years of clinical practice, y	0–5	46	25.52
	6–10	31	31.72
	11–15	14	21.38
	16–20	17	9.66
	> 20	3	11.72
Educational level	< Undergraduate	42	28.97
	≥ Undergraduate	102	70.34
Professional title	Junior	48	33.10
	≥ Intermediate	97	66.90
Practice department	Medical department	96	66.21
	Surgical department	49	33.79
Shift	Day	98	67.59
	Night	47	32.41

### Description of thestudy variables

#### Presence of interruptions and task performance

In the 145 shift observations, the average EHR task time was 84.69 (SD 56.68) minutes per shift, including 52.04 (SD 19.08) minutes of task switching and 5.60 (SD 2.79) minutes of concurrent multitasking. The total number of interruption events was 2871, indicating that the average frequency of EHR task interruptions was 14.03 times per hour. The top three sources of interruption events were as follows: nursing colleagues (1026, 35.74%), patients (773, 26.92%), and nurses themselves (366, 12.75%). Of these interruptions, 71.37% were priority events leading to task switching, while 28.63% led to concurrent multitasking. The incidence of error or near error was 158, with an average of 1.09 (SD 1.31) errors per shift, while 68.35% of errors were self-corrected by the nurses. Table 2 shows a summary of the observations of interruption events.

**Table 2:** Counts and rates of interruption sources and reactions

Sources	Interruption events (*n* = 2871) (No. %)	Task switching (*n* = 2049) (No. %)	Multitasking (*n* = 822) (No. %)
Environment	116(4.04)	97(4.73)	19(2.31)
Patients	773(26.92)	691(33.72)	82(9.98)
Patients’ family members	78(2.72)	69(3.37)	9(1.09)
Nurse colleagues	1026(35.74)	448(21.86)	578(70.32)
Nurses themselves	366(12.75)	311(15.18)	55(6.69)
Doctor	130(4.53)	90(4.39)	40(4.87)
Other clinical staff	83(2.89)	65(3.17)	18(2.19)
Technical malfunctions	77(2.68)	77(3.76)	0(0.00)
Information impediments	48(1.67)	48(2.34)	0(0.00)
Telephone/equipment alarms	106(3.69)	101(4.93)	5(0.61)
Other	68(2.37)	52(2.54)	16(1.95)

### Nurses’ mental workload, task performance and potential influencing variables

Among the 145 participants, the total mean score of the NASA-TLX was 44.57 (SD 14.08). The task performance index score was 1.09 (SD 1.31). The task difficulty index score was 33.30 (SD 6.27). The system usability score was 69.23 (SD 17.65). The professional experience score was 56.94 (SD 13.71). The self-efficacy score was 29.92 (SD 5.45). The positive effect score was 27.10 (SD 7.31), and the negative affect score was 15.65 (SD 3.81). The minimum, maximum and mean scores and standard deviations for the variables are shown in Table 3.

**Table 3:** Mean scores of mental workload, task performance and other variables (*n* = 145)

Variables	Minimum	Maximum	Mean	SD
Mental workload	6.67	80.83	44.57	14.08
Task performance	0.00	16.00	1.09	1.31
Task difficulty	11.00	48.00	33.30	6.27
System usability	20.00	90.00	56.94	13.71
Professional competency	18.00	72.00	53.92	10.94
Self-efficacy	19.00	40.00	29.92	5.45
Positive affect	10.00	50.00	27.10	7.31
Negative affect	10.00	28.00	15.65	3.81

### Hypothesis testing

#### Associationof nurses’ mental workload, performance, and potential influencing variables

The mental workload in the EHR task was associated with the task performance (*r* = 0.386, *p* < 0.001), EHR task time (*r* = 0.243, *p* < 0.001), task difficulty (*r* = 0.339, *p* < 0.001), system usability (*r* = -0.278, *p* = 0.001), and negative affect (*r* = 0.458, *p* < 0.001). The correlation coefficients between variables are presented in Table 4.

**Table 4:** Correlations of nurses’ mental workload, performance, and potential influencing variables (*n* = 145)

Variable		1	2	3	4	5	6	7	8	9	10	11	12
1	Mental workload	1											
2	Task performance	0.386^**^	1										
3	Professional title	0.089	-0.208^*^	1									
4	Task time	0.243^**^	0.306^**^	-0.113	1								
5	No. of task switching	0.148	0.180^*^	-0.186^*^	0.687^**^	1							
6	No. of multitasking	0.086	0.181^*^	-0.013	0.419^**^	0.602^**^	1						
7	Task difficulty	0.339^**^	-0.030	0.291^**^	-0.022	-0.044	0.052	1					
8	System usability	-0.278^**^	-0.069	-0.105	-0.004	-0.042	-0.034	-0.397^**^	1				
9	Professional competency	-0.050	-0.151	0.294^**^	-0.054	-0.017	-0.049	0.168^*^	0.214^**^	1			
10	Self-efficacy	-0.127	-0.046	0.199^*^	-0.096	-0.004	0.017	0.084	0.214^**^	0.669^**^	1		
11	Positive affect	-0.046	-0.082	0.076	-0.039	0.162	0.080	0.146	-0.004	0.219^**^	0.228^**^	1	
12	Negative affect	0.458^**^	0.297^**^	-0.100	0.136	0.074	0.024	0.120	-0.081	-0.065	-0.159	0.131	1

Note: ^*^ *p* < 0.05; ^**^ *p* < 0.01 (two-tailed test)

### Measurement modelling

**Fig. 1:**
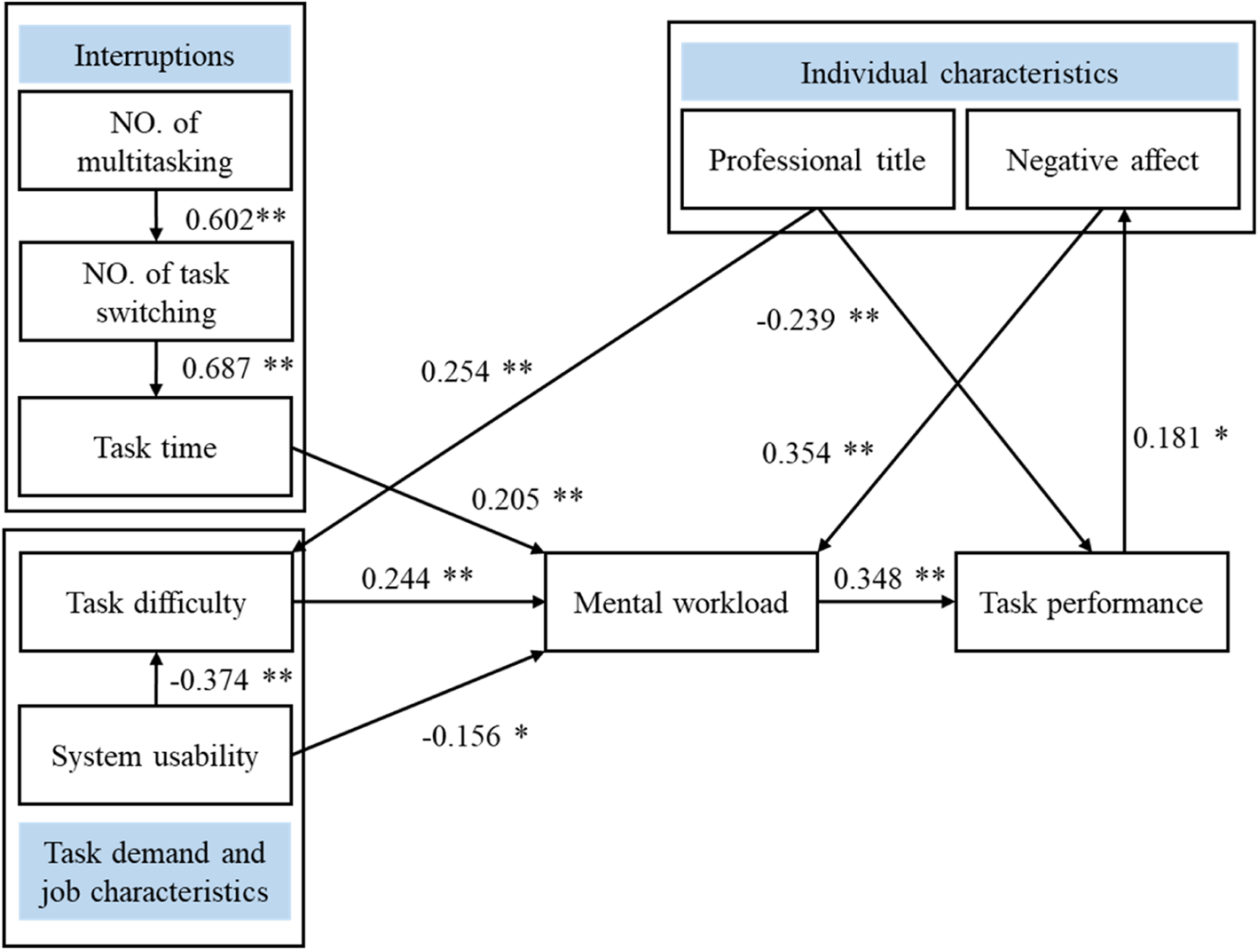
Path analysis model of influencing variables on nurses’ mental workload and performance in EHR tasks in interruptive clinical setting

A path analysis approach was employed to test the hypotheses according to the recommendations by Preacher and Hayes [42]. The overall effect of interruptions, mental workload and task performance was analysed according to hypothesis 1 (Model 1 in Tables 5 and 6). The results suggested that the EHR task time (β = -0.46, *p* < 0.001) had a significant impact on nurses’ mental workload. In addition, the mediating effect of self-efficacy on the pathway between professional experience (professional title or competency) and mental workload was tested according to hypothesis 2, however, the pathway between self-efficacy and mental workload was not statistically significant (β = -0.127, *p* = 0.125). We have modified the model 2 with variables of professional experience (professional title or competency), task difficulty, mental workload and task performance according the initial hypothesis 2 and 5 (Model 2 in Tables 5 and 6). Path analysis showed that task difficulty mediates the effect of professional title and mental workload, and professional title could influence task performance directly (β = -0.224, *p* < 0.001). Then, the relationships between negative affect and mental workload (β = 0.413, *p* < 0.001), and the relationship between task performance and negative affect (β = 0.170, *p* = 0.042) were confirmed (Model 3 in Table 5 and 6). Furthermore, the relationships between system usability and task difficulty (β = -0.397, *p* < 0.001) and mental workload (β = 0.170, *p* = 0.044) were confirmed (Model 4 in Table 5 and 6). Because of the above findings, the best fitting model including the variables professional title, task switching incidence, multitasking incidence, task time, task difficulty, system usability, negative affect, mental workload, and task performance were confirmed (Fig. 1). The fit indices of the model were χ^2^ = 30.168, *df* = 25, p = 0.218, RMSEA = 0.038, GFI = 0.924, AGFI = 0.987, CFI = 0.982, TLI = 0.974, NFI = 0.906, and IFI = 0.983 (Model A in Table 5 and 6).

**Table 5:** Model-fitting standard and fitting index of the models

Model	Description	*χ*^*2*^	*df*	*P*	RMSEA	GFI	AGFI	TLI	NFI	IFI	CFI
Model A	Full model	30.168	25	0.218	0.038	0.924	0.987	0.974	0.906	0.983	0.982
Model 1	multitasking, task switching, task time, mental workload, task performance	10.120	6	0.069	0.033	0.973	0.934	0.964	0.949	0.979	0.978
Model 2	professional title, task difficulty, mental workload, task performance	2.149	2	0.342	0.023	0.993	0.963	0.993	0.967	0.998	0.998
Model 3	negative effect, mental workload, task performance	3.145	2	0.076	0.122	0.986	0.915	0.888	0.948	0.964	0.963
Model 4	task difficulty, system usability, mental workload	5.112	2	0.078	0.104	0.983	0.914	0.864	0.932	0.957	0.955

**Table 6:** Estimates of the standardized regression weights of the models

Significant path	Model A	Model 1	Model 2	Model 3	Model 4
task switching < – multitasking	0.602***	0.602***			
task time < – task switching	0.687***	0.687***			
mental workload < – task time	0.205*	0.243**			
task performance < – mental workload	0.348***	0.386***	0.408***	0.325***	0.386***
task performance < – professional title	-0.239***		-0.224***		
task difficulty < – professional title	0.254***		0.291***		
mental workload < – task difficulty	0.244***		0.339***		0.272***
mental workload < – negative effect	0.354***			0.413***	
negative effect < – task performance	0.181*			0.170*	
task difficulty < – system usability	-0.374***				-0.397***
mental workload < – system usability	-0.156*				-0.170*

Note: * *p* < 0.05, ** *p* < 0.01, ***p < 0.001 (two-tailed test)

## Discussion

### Key findings

This study identified three important findings. First, to our knowledge, this is the first study to provide a description of a large number of EHR task interruptions. The findings of this study showed that the magnitude of interruption in EHR tasks was related to the incidence of errors and near errors; fortunately, 68.35% of errors could be corrected by nurses themselves. Second, the study highlighted the two major reactions of nurses facing an EHR task interruption, namely, task switching and concurrent multitasking, of which task switching was related to task performance. Third, this study employed a mental workload theoretical framework to explore the multilevel factors associated with nurses’ mental workload and task performance, that is, interruptions, task time, nurses’ working experience, EHR usability, task difficulty and negative affect, providing a comprehensive understanding of the cognitive process in EHR tasks in dynamic clinical settings.

### Interruption in EHR tasks and its relationship with mental workload and performance

In our study, the average frequency of interruptions was 14.03 times per hour, while relevant studies have reported an average of 8.13 interruptions per hour in medication administration [43] and an average of 5.40 to 10.90 interruptions per hour in nursing care in emergency departments [6, 44]. Although there is a lack of studies on interruptions in EHR tasks, the interruption incidence found for EHR tasks in this study was much higher than that of other nursing procedures or general daily nursing care. This is explainable, as EHR tasks are considered the lowest priority compared with patients’ requirements and medical orders [2], which is why we observed a proportion of nurses performing EHR tasks in their off-hours. Our finding calls for attention to the high incidence of interruptions, as longer task time caused by interruptions contributes to higher mental workload and thus to increased task errors in our study.

In this study, we confirmed that task switching is far more frequent than concurrent multitasking in EHR tasks. Task switching occurs when nurses face human interaction, such as treatment requirements from patients and interpersonal communication, or when the interruption is a task that requires a larger amount of time (with an average duration of 1.53 min); on the other hand, multitasking tends to occur with shorter tasks with an average time of 0.92 min and is more likely to occur when nurses engage in simple communication. As the path analysis model indicates a linkage from concurrent multitasking and task switching to task time, the incidence and duration of the interruption seems to be one of the major causes for mental workload. This could be explained by the fact that when the nurses are interrupted in their task, their attention is shifted from the initial process to the new process with a decay of the memory of the initial process; this leads to an increase in response time when returning to the initial task or even the nurse starting the initial task over and hence a likelihood of overwhelmed cognitive capacities [14] and decreased productivity and accuracy in the EHR task [45, 46].

### Multilevel predictors of mental workload and performance in EHR tasks

Apart from interruptions, we also measured other factors, including task difficulty, system usability, professional experience and competency, and self-efficacy according to the human factors and ergonomics perspective of mental workload [13], to describe cognitive processes and explore the predictors of mental workload and performance in EHR tasks. The total mean mental workload score in EHR tasks was 46.21 (SD 14.76), indicating a medium level of mental workload. Regarding objective workload, the net duration of EHR task time was an average of 32.56 min per shift, which was close to the restriction (30 min) on shift task hours by the National Health Commission of the People's Republic of China [47]. However, the average total time spent on EHR tasks was 84.69 (SD 56.68) minutes per shift due to the high number of interruption events, which indicates that interruptions could influence task efficiency, leading to a higher level of temporal demands. In addition, the task difficulty index (33.30 SD 6.27) reflected a moderate to high complexity and difficulty of EHR tasks, contributing to a higher self-reported level of cognitive demands. Hence, it is reasonable to suggest that task difficulty is a predictor of mental workload, and this result is consistent with other studies [48, 49].

In our study, the path analysis indicated that task difficulty is partially affected by professional title. This finding may be due to the role of nurses in the study hospital, with those with higher professional titles tending to be in charge of complicated cases. In addition, professional title is a protective factor for task performance in the path analysis model. In this study, professional experience was reflected by two variables: professional title and competency. We did not verify that professional competency was associated with mental workload or task performance. It is reasonable that objective indicators would be far more accurate than subjective indicators to reflect real professional experience because participants may exaggerate their competency in a self-report scale. One reason why professional title was related to task performance but not to mental workload might be that experienced nurses will ask for help from colleagues when necessary, thus reducing their error and mental workload, while novices may not realize their mistakes; thus, errors occurred without the elevation of mental workload [21]. Coincidentally, the observation showed that concurrent multitasking events generally involved interactions with colleagues, among which most were problem-solving interactions. Hence, interruptive events may also be beneficial and necessary for the quality of the task [4].

The usability of EHR system is a major concern in modern nursing care, and suboptimal usability has been proven to be associated with clinician burnout and patient safety events [26]. In our study, the total mean system usability score was 56.94 (SD 13.71), indicating a medium level of reported usability. Even though the investigated hospital employs one of the most intelligent EHR systems in China, which allows offices to be paperless, information overload and information conflict were the major complaints in the post-observation conversations with participants. This result seems to be in line with findings in the study from Beasley and colleagues [50]. In our study, system usability was associated with mental workload directly or mediated by task difficulty, which is consistent with the finding in a simulated scenario test and could be explained by the fact that suboptimal EHR usability is associated with the elevation of task effort [26]. Therefore, organizations should develop explicit policies and procedures for enhancing EHR usability.

Moreover, the results of this study showed that task performance and mental workload interacted with each other, which is consistent with ergonomic studies [51]. Given the effect of mental workload on performance, insufficient stimulation is known to lead to underload, boredom, and decreased performance [52]. Conversely, overload is also known to decrease task performance [53]. Therefore, the relationship between mental workload and performance is curvilinear, while in this study, it was linear. This might be because the range in mental workload in our study was approximately moderate to high, which reflects only half of the curvilinear model. Another possible reason is that this level of mental workload might be appropriate for good working performance, which is well illustrated in the mental workload model of Hancock and Chignell [51, 52]. Regarding the effect of performance on mental workload [54], perceived performance, especially errors, influences mental workload by requiring more information processing resources and elevating emotional workload [55]. Hence, the model presents a trend in that the more errors that occur, the higher the negative affect will be.

In our study, the total mean self-efficacy score for EHR tasks was 29.92 (SD 5.45), and the range was at a high level, meaning that the majority of participants believed they were confident and competent in performing EHR tasks. However, this variable was not included in the path analysis model. According to the technology acceptance model [22], which is one of the most common social cognitive theories used in behavioural research on health care professionals, social, individual and contextual factors interact with each other to predict behaviours related to EHR tasks, among which individual factors generally encompass variables such as self-efficacy and professional experience [56]. Hence, the typical variable selection and hypothetical model in our study was reasonable. The exclusion of self-efficacy could indicate that in terms of individual variables, professional experience is far more important for positive EHR task behaviour than self-efficacy. It is obvious that knowledge, skills and situational awareness are essential for quality practice and for developing self-efficacy [6].

### Strengths and limitations points

To our knowledge, this is the first study showing and describing the magnitude of nursing workflow interruptions in electronic health record tasks. This study employed a mental workload theoretical framework to explore the multilevel factors associated with nurses’ mental workload and task performance in real-world clinical settings. Nevertheless, several limitations of this study should be noted in the interpretation of our findings. First, we conducted a real-world study by employing the core variables from the theoretical framework of mental workload to explore a predictive model for EHR task cognition and behaviour; however, mental workload was statically assessed, which may have involved recall bias and limited inferences concerning the dynamic problems that evolve over time in EHR tasks. Future studies should employ objective and longitudinal evaluation tools to reflect multilevel variables and provide a coherent picture of flexible adaptation to dynamic care settings. In addition, the technique of non-participatory observation may have modified participants’ behaviour under the data collection conditions even though the observer established a good cooperative relationship with participants. Since the observer acted as a research tool, fatigue due to the long observation duration may have led to the neglect of some important clinical information. Although the observer was knowledgeable and well trained in EHR tasks, real-time error recognition is limited, especially in this kind of environment with a fast working pace.

### Relevance to clinical practice

Based on the study findings, reducing harmful interruptions to decrease task time can avoid negative outcomes. Nursing leaders should comprehensively understand the characteristics of interruptions in EHR tasks, which are contextual, multifarious, complex and dynamic, to develop targeted interventions. Training nurses to cope with interruptions and mitigate the impact of interruptions has the potential to decrease nurses’ mental workload and improve task performance. As perceived task difficulty is one of the key variables influencing nurses’ mental workload, which shows differences in work experiences, and EHR usability has been found to induce task difficulty and mental workload, sufficient training related to EHR implementation and task operation would be particularly important in nursing management. Moreover, improving system usability is beneficial to nurses to reduce mental workload and increase the efficiency and accuracy of EHR tasks.

## Conclusions

Our study employed an observational approach in a naturalistic clinical practice setting and provided the first empirical evidence that the magnitude of workflow interruptions in EHR task could increase nurses’ mental workload and impair their task performance. By drawing upon a theoretical framework from ergonomics, multilevel predictors of mental workload and task performance in EHR tasks, including task time, task difficulty, system usability, working experience and negative affect, were confirmed, which provided support for targeted quality improvement programs.

## Supplementary Information

**Additional file 1.** Observational tool for EHR Tasks.

**Additional file 2: Appendix Figure 1.** Hypothetical framework of influencingvariables on nurses’ mental workload and performance in EHR tasks ininterruptive clinical setting.

## Data Availability

The data sets used for the current study are available from the corresponding author upon request.

## Abbreviations

EHR: Electronic health record
NASA-TLX: National Aeronautics and Space Administration Task Load Index
RMSEA: Root mean square error of approximation
GFI: Goodness-of-fit index
AGFI: Adjusted goodness-of-fit index
CFI: Comparative fit index
TLI: Tucker-Lewis index
NFI: Normed fit index
IFI: Incremental fit index
