# Supplementary material for: Workflow interruption and nurses’ mental workload in electronic health record tasks: An observational study
Source: BMC Nurs. 2023 Mar 9;22:63. doi: 10.1186/s12912-023-01209-9 (PMC9996908; doi:10.1186/s12912-023-01209-9)
Supplement: Supplementary file 1 — Additional file 1. Observational tool for EHR Tasks. [file 12912_2023_1209_MOESM1_ESM.docx]

**Observational tool for** **EHR Tasks**

**Sociodemographic Questionnaire**

**Please Fill in Your Personal Information According to the Following Table:**

| 1 | **Gender** □ Man _1_ □ Woman _2_ |
| --- | --- |
| 2 | **Age** years |
| 3 | **Hospital Location**： City ( County), Province |
| 4 | **Hospital Level**：□ Tertiary _3_ □ Secondary _2_ □ Primary _1_ |
| 5 | **Hospital Informatization Level**：□ 6 _6_ □ 5 _5_ □ 4 _4_ □ 3 _3_ □ 2 _2_ □ 1 _1_ |
| 6 | **Marriage Status**：□ Married _1_ □ Unmarried _2_ |
| 7 | **Years of Clinical Practice**： years |
| 8 | **Practice Department**： |
| 9 | **First-Record of College**：□ Secondary _1_ □ College _2_ □ Bachelor _3_ □ Master _4_ □ Doctor _5_ |
| 10 | **Highest Education**：□ Secondary _1_ □ Associate _2_ □ Bachelor _3_ □ Master _4_ □ Doctor _5_ |
| 11 | **Professional Title**：□ Nurse _1_ □ Senior Nurse _2_ □ Supervisor Nurse _3_ □ Co-Chief Nurse _4_ □ Chief Nurse _5_ |
| 12 | **Average Time of Processing Electronic Health Record System Per Day Last Month**： Hours |

**Summary of EHR System Task Observations**

**Observation Site： Start Time: End Time: Total duration of interruption：**

| **Tasks** | □ Patient Data Entry _1_ | □ Nursing Evaluation _2_ | □ Medical Record Query _3_ | | □ Nursing Plan/Course _4_ | | □ Discharge Planning _5_ | |
| --- | --- | --- | --- | --- | --- | --- | --- | --- |
| **Shift and Role** | □ Office Shift _1_ | □ Primary Nursing Shift _2_ | □ Treatment Shift _3_ | | □ Other Shifts _4_ | |  | |
| **Number of Patients in the Task** |  | **Nursing responsibility** | □ Primary _1_ | | □ Function _2_ | | Others _3_ ( ) | |
| **Computer System** | Windows , G, Bit. | | | | | | | |
| **EHR System** |  | | | | | | | |
| **Onlookers** | □ Patients _1_ | □Patients’ Family Members _2_ | □Colleagues _3_ | □Students _4_ | | □Supervisors _5_ | | □None _0_ |
| **Number of Nurses per Shift** |  | **Number of Patients in Charge** |  | | **Number of Patients in the Ward** | |  | |
| **Noise** | **dB** | **How Noisy the Environment is When You Finish This Task?**   \| 0 \| 1 \| 2 \| 3 \| 4 \| 5 \| 6 \| 7 \| 8 \| 9 \| 10 \| \| --- \| --- \| --- \| --- \| --- \| --- \| --- \| --- \| --- \| --- \| --- \| | | | | | | |
| **Light** | □ Suitable _1_ | □ Unsuitable _2_ | **Temperature** | | □ Suitable _1_ | | □ Unsuitable _2_ | |

**Summary of Task Switching during EHR tasks**

| **Number** | **Start / End Time** | **Source** | **Content** | **Perception** | **HR (bpm)** | **Noise (dB)** | **Remarks** |
| --- | --- | --- | --- | --- | --- | --- | --- |
| 1 |  | Environment ;  Number of Times ( ) | 1. Related to the Current Case;  2. Related to other Current Cases;  3. Related to Completed Cases;  4. Related to New Cases;  5. Related to Organization and Coordination;  6. Related to Patient Comfort;  7. Others. |  |  |  |  |
| 2 |  | Nurses’ Colleagues ;  Number of Times ( ) |  |  |  |  |  |
| 3 |  | Patients ;  Number of Times ( ) |  |  |  |  |  |
| 4 |  | Patients’ Family Members ;  Number of Times ( ) |  |  |  |  |  |
| 5 |  | Nurses Themselves ;  Number of Times ( ) |  |  |  |  |  |
| 6 |  | Doctors ;  Number of Times ( ) |  |  |  |  |  |
| 7 |  | Other Clinical Staff ;  Number of Times ( ) |  |  |  |  |  |
| 8 |  | Clinical Support Personnel ;  Number of Times ( ) |  |  |  |  |  |
| 9 |  | Telephone/Equipment Alarms ;  Number of Times ( ) |  |  |  |  |  |
| 10 |  | Technical Malfunctions ;  Number of Times ( ) |  |  |  |  |  |
| 11 |  | Information Impediments ;  Number of Times ( ) |  |  |  |  |  |
| 12 |  | Clinical Teaching ;  Number of Times ( ) |  |  |  |  |  |
| 13 |  | Others .  Number of Times ( ) |  |  |  |  |  |

**Summary of Incidence of Errors and Near Errors during EHR tasks**

| **Number** | **Errors and near errors** | **Corrected (Yes or No)** | **How to Correct** |
| --- | --- | --- | --- |
| 1 |  | □ Yes 1 □ No 0 |  |
| 2 |  | □ Yes 1 □ No 0 |  |
| 3 |  | □ Yes 1 □ No 0 |  |
| 4 |  | □ Yes 1 □ No 0 |  |
| 5 |  | □ Yes 1 □ No 0 |  |
| 6 |  | □ Yes 1 □ No 0 |  |
| 7 |  | □ Yes 1 □ No 0 |  |

**Summary of Concurrent Multitasking during EHR tasks**

| **Number** | **Start / End Time** | **Source** | **Purpose** | **Type** | **Nurses’ Reaction** | **HR (bpm)** | **Noise (dB)** | **Remarks** |
| --- | --- | --- | --- | --- | --- | --- | --- | --- |
| 1 |  | Environment ;  Number of Times ( ) | 1. Acquire Knowledge or Information;  2. Receiving and Making Calls;  3. Improper Preparation, Replacement of Instruments, etc.;  4. Instrument Failure, Check and Replace the Instrument;  5. Teaching/Supervision;  6. Resolve Alarms or Calls. | 1. Current Medical Tasks;  2. Other Medical Tasks;  3. Non-Medical Tasks. |  |  |  |  |
| 2 |  | Nurses’ Colleagues ;  Number of Times ( ) |  |  |  |  |  |  |
| 3 |  | Patients ;  Number of Times ( ) |  |  |  |  |  |  |
| 4 |  | Patients’ Family Members ;  Number of Times ( ) |  |  |  |  |  |  |
| 5 |  | Nurses Themselves ;  Number of Times ( ) |  |  |  |  |  |  |
| 6 |  | Doctors ;  Number of Times ( ) |  |  |  |  |  |  |
| 7 |  | Other Clinical Staff ;  Number of Times ( ) |  |  |  |  |  |  |
| 8 |  | Clinical Support Personnel ;  Number of Times ( ) |  |  |  |  |  |  |
| 9 |  | Others .  Number of Times ( ) |  |  |  |  |  |  |

**Observation Form for Operation Steps (Contents) of EHR Tasks**

| **Tasks** | **Start / End Time** | **People, events, influencing factors/errors** | **Causes** | **Measures to Reduce Errors** |
| --- | --- | --- | --- | --- |
| **Nursing Medical Record** | | | | |
| 1. Admission Evaluation List |  |  |  |  |
| 2. Summary of Admission Nursing Evaluation |  |  |  |  |
| 3. Record of First Nursing |  |  |  |  |
| 4. General Nursing Record |  |  |  |  |
| 5. TCM Nursing Operation Record Form |  |  |  |  |
| 6. Critical Care Record |  |  |  |  |
| 7. Health Education Registration Form |  |  |  |  |
| **Nursing Evaluation** | | | | |
| 1. Patient Care Grading Form |  |  |  |  |
| 2. Pain Assessment Record |  |  |  |  |
| 3. Nutrition Risk Screening Record |  |  |  |  |
| 4. Risk Factors Assessment and Report Form of Stress Injury |  |  |  |  |
| 5. Fall Prediction Evaluation Form |  |  |  |  |
| 6. Catheter Risk |  |  |  |  |
| 7. Risk Assessment of Pulmonary Embolism |  |  |  |  |
| 8. Transport Form |  |  |  |  |
| 9. Informed Consent |  |  |  |  |
| 10. Discharge Guidance |  |  |  |  |
