# Supplementary material for: Workflow interruption and nurses’ mental workload in electronic health record tasks: An observational study
Source: BMC Nurs. 2023 Mar 9;22:63. doi: 10.1186/s12912-023-01209-9 (PMC9996908; doi:10.1186/s12912-023-01209-9)
Supplement: Supplementary file 2 — Additional file 2: Appendix Figure 1. Hypothetical framework of influencingvariables on nurses’ mental workload and performance in EHR tasks ininterruptive clinical setting. [file 12912_2023_1209_MOESM2_ESM.docx]

**
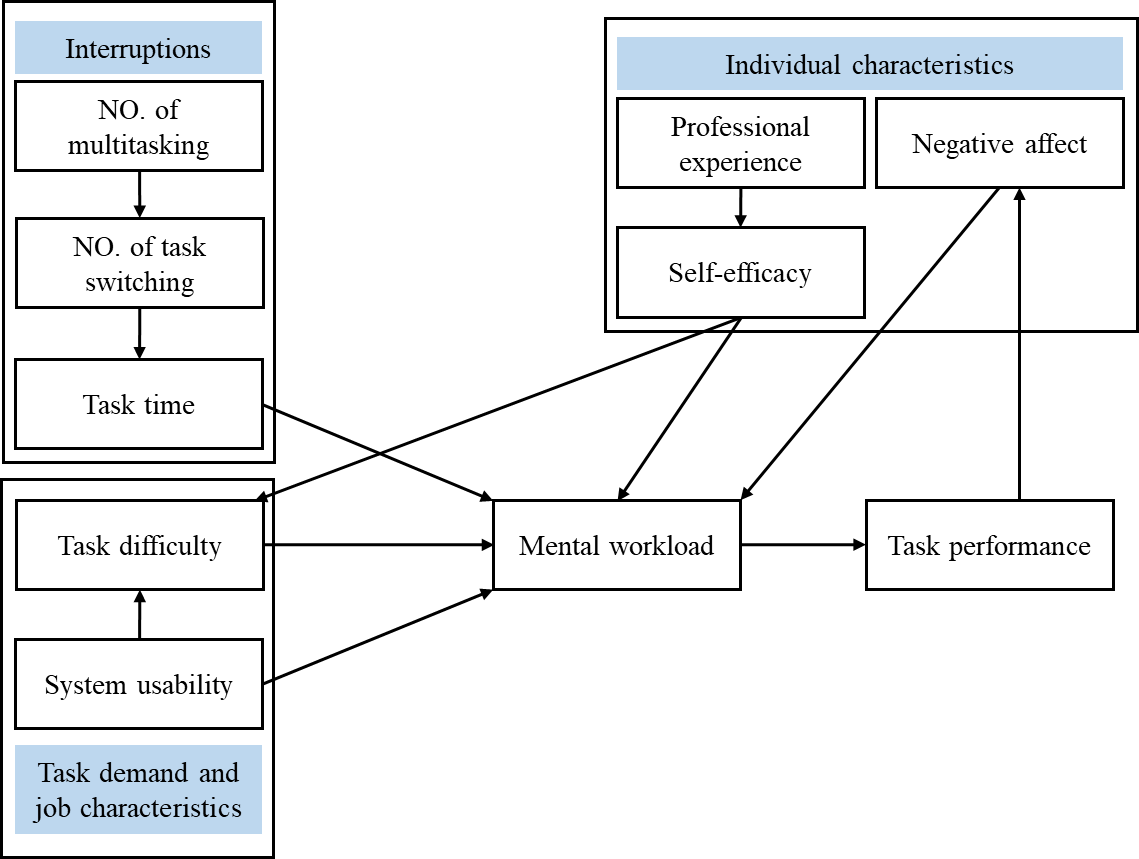
**

**Appendix Figure 1 Hypothetical framework of influencing variables on nurses’ mental workload and performance in EHR tasks in interruptive clinical setting**
